# Plasticity of feeding behaviour traits in response to production environment (temperate vs. tropical) in group-housed growing pigs

**DOI:** 10.1038/s41598-021-04752-0

**Published:** 2022-01-17

**Authors:** Nausicaa Poullet, Wendy M. Rauw, David Renaudeau, Juliette Riquet, Mario Giorgi, Yvon Billon, Hélène Gilbert, Jean-Luc Gourdine

**Affiliations:** 1 URZ, INRAE, 97170 Petit-Bourg, Guadeloupe France; 2 Departamento de Mejora Genética Animal, INIA-CSIC, Ctra de la Coruña km 7.5, 28040 Madrid, Spain; 3 PEGASE, INRAE Agrocampus Ouest, 35590 St Gilles, France; 4 GenPhySE, Université de Toulouse, INRAE, INP, 31320 Castanet-Tolosan, France; 5 PTEA, INRAE, 97170 Petit-Bourg, Guadeloupe France; 6 GenESI, INRAE, 17700 Surgères, France

**Keywords:** Animal breeding, Animal behaviour, Genetic interaction

## Abstract

Heat stress affects pig metabolism, health and welfare, resulting in reduced growth and important economic losses. The present experiment aimed to evaluate the effects of two climatic environments [temperate (TEMP) vs. tropical humid (TROP)] on feeding behaviour in growing pigs. The feeding behaviour traits were measured with automated feeders and included: daily feed intake, daily eating time, feeding rate, daily number of meals, feed intake per meal, and feeding time per meal. Pigs came from a backcross population between Large White (LW, heat sensitive) and Creole (CR, heat tolerant) pigs. The same 10 F1 LW × CR boars (sire families [SF]) were mated with related LW sows in each environment. Feeding behaviour was recorded for a total of 1,296 pigs (n = 634 pigs for TEMP and n = 662 pigs for TROP) between 11 and 23 weeks of age. Growth performance and thermoregulatory responses (rectal and skin temperatures) were also measured. Results show that TROP conditions affect feeding behaviour traits: animals had more meals per day but these meals were smaller both in duration and in size, resulting in lower daily feed intake and less time eating per day. Significant SF by environment (GxE) interactions were found for all feeding behaviour traits. When SF were distributed into robust and sensitive groups (previously defined according to performance and thermoregulatory traits), results showed group by environment interactions for all feeding traits, except meal frequency. Moreover, a significant difference in feeding rate between robust and sensitive group was detected in TEMP, suggesting that feeding rate may be a good candidate to evaluate heat tolerance.

## Introduction

High temperatures are responsible for important economic losses for the pig industry, both in tropical regions where average ambient temperatures often exceed 25 °C but also in temperate regions that are confronted with more frequent heat waves during the summer months^1^. Pig performance is reduced during Heat Stress (HS) mainly due to their reduced voluntary feed intake as a mean to lower metabolic heat production^2,3^. Therefore, HS is becoming an essential issue for pig production and in recent years, many research studies have focused on the impact of HS on swine metabolism and physiology (reviewed in Belhadj Slimen et al*.*^4^ and Ross et al*.*^5^). However, apart from the overall reduction of feed intake with high temperature, very little information is available on the effect of HS on pig feeding behaviour. Several studies have demonstrated strong phenotypic and genetic correlations between feeding behaviour and performance traits^6–8^. For instance, feeding rate is positively phenotypically correlated with daily feed intake (0.29), but also with average daily gain (ADG, 0.40) and fat deposition (0.28), meaning that pigs that eat faster, eat more and grow faster and fatter^8^. Similar trends were found for genetic correlations in Large White pigs: feeding rate was positively correlated to ADG (0.48) and negatively correlated to carcass lean content (0.24)^7^. Investigating plasticity of feeding behaviour—i.e. how animals modify their feeding behaviour—in response to HS, could therefore allow a better understanding of the mechanisms responsible for reduced feed intake during HS and eventually help to redesign feeding programs to reduce the negative impacts of HS. For instance, a recent study showed that changing feeding times to the night decreased the detrimental effects of HS on lactating sows^9^.

Feeding behaviour is easily accessible through the use of automatic feeders and can be defined by criteria such as daily eating time, number of meals per day, duration and size of each meal and feeding rate. These feeding behaviour traits are highly plastic and vary with the physical environment of pigs, e. g. space allowance, group size, flooring conditions, presence of enrichment and temperature^10^. Previous results suggest that reduction of feed intake during HS may occur through the reduction of meal size rather than number of meals^11,12^. The animal response to HS is highly variable both within and between populations, and part of this variability may have a genetic basis^13–15^. Evaluating GxE interactions for feeding behaviour, i.e. how different genotypes change their feeding behaviour in response to HS, is essential to understand how heat tolerance translates in terms of feeding behaviour and may provide potential phenotypic markers to include in selection for heat tolerance. Here, we used a backcross population between Large White (LW, heat sensitive) and Creole (CR, heat tolerant) to 1) evaluate the effects of tropical humid conditions [TROP] on feeding behaviour traits, focusing on the interactions between sire family and environment (GxE) and 2) estimate phenotypic relationships between feeding behaviour, performance and thermoregulation traits in temperate (TEMP) and TROP conditions.

## Results

### Animal and climatic characteristics

**Figure 1:**
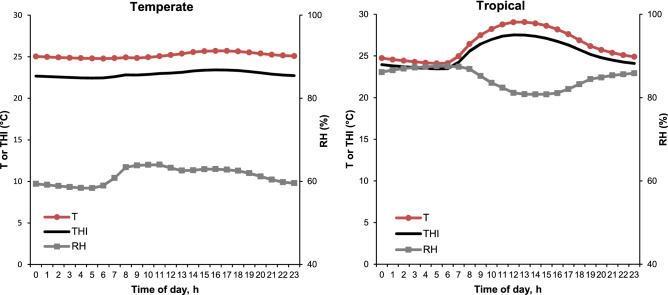
Hourly climatic variation in the pig building accrording to the production environment (temperate or tropical). T = ambient temperature, RH = relative humidity, THI = temperature-humidity index, calculated according to the following formula: THI (°C) = T − (0.55 − 0.0055 × RH) × (T − 14.5), proposed by the National Oceanic and Atmospheric Administration^49^, cited by Zumbach et al*.*^50^.

We previously showed with the same experimental design that the Temperature-Humidity Index (THI) had a significant difference of 2.4 °C between TEMP and TROP^16^. Figure 1 shows the hourly variation of temperature (T), relative humidity (RH) and THI in TEMP and TROP experimental facilities. In TEMP conditions, the daily variations of THI were small, between 22.4 °C at 0500 h and 23.4 °C at 1600 h, whereas in TROP environment, variations in THI were stronger, with minimum and maximum values reached at 0500 h (23.4 °C) and 1200 h (27.5 °C), respectively. All production and thermoregulation traits were significantly affected by the environment (P < 0.05)^16^. The ADG between 11 and 23 week of age was lower in TROP compared to TEMP (751 vs. 833 g/d, P < 0.001). Final Body Weight (BW) and BackFat Thickness (BFT) were lower in TROP than in TEMP conditions (84.9 vs. 103.4 kg and 15.6 vs. 20.6 mm, P < 0.001). Both feed efficiency measures, Feed Conversion Ratio (FCR) and Residual Feed Intake (RFI), were lower in TROP than in TEMP climates (2.39 vs. 2.69 kg feed/kg BW gain, P < 0.001 and − 29.2 vs. 16.8 g/d, P < 0.05), indicating that animals were more efficient in TROP. For all thermoregulatory body measures (Rectal Temperature [RT], Skin Temperature [ST]), greater values were found for pigs in TROP than in TEMP (on average 35.9 vs 34.8 °C for ST, and 39.5 vs 39.3 °C for RT, respectively, P < 0.05).

### Environmental effects on feeding behaviour traits

All feeding behaviour traits were significantly affected by the environment (P < 0.05, Table 1). In TROP, animals had reduced Average Daily Feed Intake (ADFI; 1.79 vs. 2.24 kg/d, P < 0.001), reduced daily eating time (66.9 vs. 74.2 min/d, P < 0.001) and reduced feeding rate (28.7 vs. 33.9 g/min, P < 0.001) compared to TEMP. The lower ADFI in pigs in TROP was associated with a decrease of meal size (253 vs. 386 g/meal, P < 0.001) and duration (9.4 vs. 12.7 min/meal, P < 0.001), but with an increased number of meals (7.5 vs 6.1 meals/d, P < 0.001).

**Table 1:** Least squares means effect of production environment, sire family and sex on feeding behaviour traits.

	Environment		RSD^1^	Significant effects^2^
	Temperate	Tropical		
Number of pigs	634	662		
Mean components of feeding behaviour				
ADFI^3^, g/d	2,240	1,792	423	SF***, E***, S***, Pe***, SFxE***
Daily eating time, min/d	74.2	66.9	15.2	SF***, E***, S***, Pe***, SFxE***
Feeding rate, g/min	33.9	28.7	7.92	SF***, E***, Pe***, SFxE***
Number of visits	28.8	21.7	14.9	SF***, E***, S***, Pe^†^
Number of meals	6.1	7.5	1.7	SF***, E***, S**, Pe***, SFxE*
Characteristics of the meals				
Meal size, g	386	253	94	SF***, E***, S*, Pe***, SFxE***
Meal duration, min	12.7	9.4	3.0	SF***, E***, Pe**, SFxE*

^1^Residual Standard Deviation.

^2^From an analysis of variance with a linear model including the effects of Sire Family (SF), Environment (E), Sex (S), Period of feed intake recording (Pe) and their interactions as fixed effect. Batch within environment was significant for all traits and is not reported in the table. Statistical significance: ***P < 0.001, **P < 0.01, *P < 0.05, ^†^P ≤ 0.10.

^3^ADFI = Average Daily Feed Intake.

**Figure 2:**
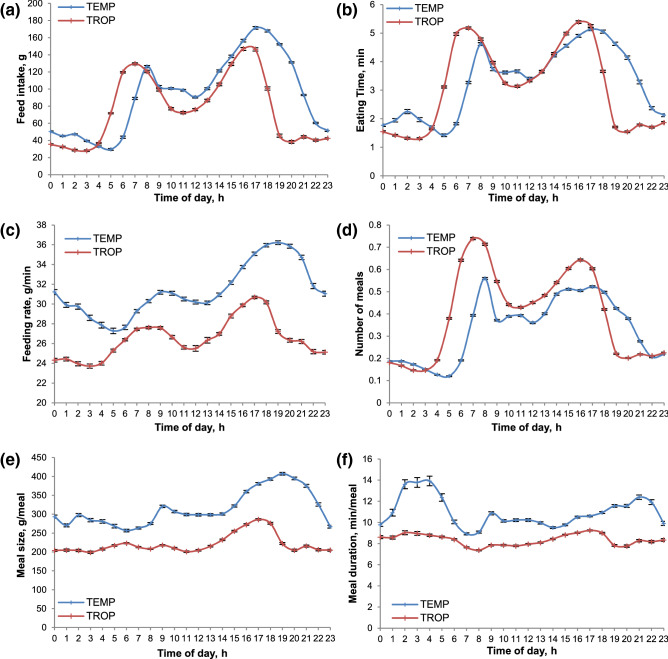
Hourly distribution of feeding parameters according to the production environment. Temperate (red, n = 634), tropical (blue, n = 662). (**a**) Feed intake, (**b**) time feeding, (**c**) feeding rate, (**d**) number of meals, (**e**) feed intake per meal, (**f**) time per meal. Error bars represent standard deviation.

Hourly feed intake peaked twice a day as illustrated in Fig. 2a. On average, the first and the second peaks were observed between 04:00 and 10:00 and between 13:00 and 21:00, respectively. The daily kinetics of hourly feed intake was affected by environment (P < 0.001) with TROP animals having their peak of consumption earlier in the morning and with lower feed intake throughout the day. When the circadian rhythm of FI was compared with the daily kinetics of the other feeding parameters, the morning peak of FI coincided with the peak of frequency of meals and time spent eating (Fig. 2b, d), whereas the afternoon peak corresponded to the fastest feeding rate, largest meal size and a lower peak of meal frequency (Fig. 2c–f). Similar to the circadian rhythm of FI, the daily kinetics of all feeding behaviour traits was affected by environment (P < 0.001), the main difference between TROP and TEMP being a shift in the morning and afternoon peaks which occur on average 2 h earlier in TROP, except for meal duration where no peak was observed in TROP.

A significant effect of sex (females vs. castrated males), was also observed for most feeding behaviour traits (P < 0.05), except for feeding rate and meal duration (P > 0.10). However, focus of the study was not put on the effects of sex, but rather on the effect of the environment and GxE interactions. In terms of differential response to the environment, we did not find significant interactions between sex and environment, suggesting that there is no difference between males and castrated females in their feeding behavior response to TROP conditions.

### Correlations between feeding behaviour traits

**Figure 3:**
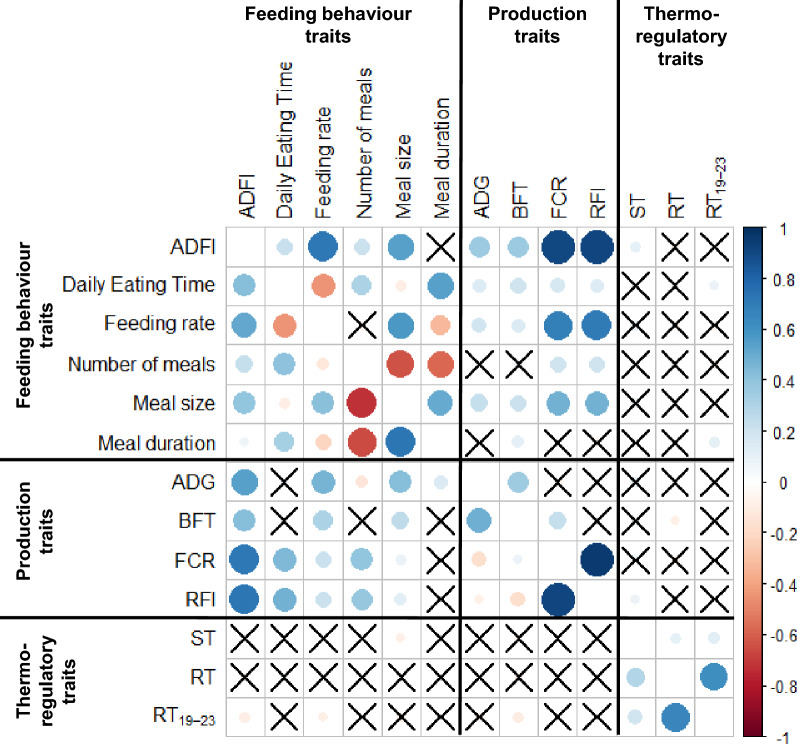
Pearson residual correlation plot between feeding behaviour and production traits. Above the diagonal: correlations between traits measured in temperate environment; below the diagonal: correlations between traits measured in tropical environment. The size of the circle is proportional to the absolute value of R coefficient of correlation of Pearson. The significance of correlation is P < 0.05 (Pearson correlation test) and the crosses indicate non-significant P values (P > 0.05). ADFI = Average Daily Feed Intake, ADG = Average Daily Gain between 11 and 23 weeks of age, BFT = Backfat Thickness at 23 week of age, FCR = Feed Conversion Ratio between 11 and 23 weeks of age, RFI = Residual Feed Intake between 11 and 23 weeks of age, ST = Skin Temperature, RT = Rectal Temperature.

Significant residual correlations were found between feeding behaviour traits in each environment (P < 0.05, Fig. 3). Similar trends of correlations were found in each environment. In both conditions, ADFI was positively correlated with daily eating time, number of meals and meal size (r = 0.21, r = 0.21, r = 0.54 respectively in TEMP and r = 0.42, r = 0.23, r = 0.39 respectively in TROP, P < 0.001). Number of meals was negatively correlated with meal size and duration (r =  − 0.64, r =  − 0.57, respectively in TEMP and r =  − 0.73, r =  − 0.66, respectively in TROP, P < 0.001), and positively correlated with daily eating time (r = 0.32 in TEMP and r = 0.40 in TROP, P < 0.001). Feeding rate was positively correlated with meal size (r = 0.58 in TEMP and r = 0.42 in TROP; P < 0.001), and negatively correlated with daily eating time and meal duration (r =  − 0.46, r =  − 0.33, respectively in TEMP and r =  − 0.44, r =  − 0.22, respectively in TROP; P < 0.001). Few traits vary importantly in their correlation coefficient between TEMP and TROP conditions. Residual correlation between feeding rate and ADFI was higher in TEMP than TROP (r = 0.71 vs. 0.51, P < 0.001). There was a higher residual correlation between meal size and meal duration in TROP than in TEMP (r = 0.72 vs. r = 0.50, P < 0.001 respectively).

### Phenotypic correlation between behaviour traits and production traits

Similarly to residual correlations between feeding behaviour traits, similar trends in both environments were found for residual correlations between feeding behaviour and production traits (Fig. 3). In both environments, feed efficiency measures (FCR and RFI) were highly correlated to ADFI (r = 0.90, r = 0.91 in TEMP and r = 0.72, r = 0.73 in TROP, P < 0.001). Nevertheless, strong differences in the size of correlations were found between environments. In particular, residual correlation between feeding rate and ADG were higher in TROP than in TEMP (r = 0.46 vs. 0.19, P < 0.001). FCR and RFI showed high correlation with daily eating time in TROP (r = 0.45, r = 0.48 respectively, P < 0.001) whereas in TEMP, FCR and RFI were more strongly correlated with feeding rate (r = 0.67, r = 0.70 respectively, P < 0.001) and meal size (r = 0.47, r = 0.47 respectively, P < 0.001).

### Phenotypic correlation between behaviour traits and thermoregulation traits

In both environments, thermoregulation traits were poorly correlated with feeding behaviour traits and these correlations were rarely significant (P > 0.05, Fig. 3). ST showed a small positive correlation with ADFI in TEMP only (r = 0.10, P < 0.01). Mean rectal temperature between 19 and 23 weeks (RT_19-23_) was positively correlated with daily eating time and meal duration in TEMP (r = 0.09, P < 0.05; r = 0.12 respectively, P < 0.01), whereas in TROP it was mostly correlated with ADFI and feeding rate (r =  − 0.09, P < 0.05 and r =  − 0.08, P < 0.05 respectively).

### Sire Family × environment effects on feeding behaviour

The effect of the interactions between sire family and environment was significant for all feeding behaviour traits (Table 1, P < 0.05). In a previous paper with the same dataset^16^, assessment of each SF sensitivity to HS was performed according to 5 production and thermoregulatory traits and showed that families 7, 1 and 2 were the most robust sire families, i.e. showing the less variation in the 5 traits of interest between the two climates, whereas families 6, 5 and 10 were the most sensitive. The robust and sensitive SF were grouped in robust and sensitive group, respectively, and the feeding behaviour of their progeny was compared (Table 2). All feeding behaviour traits, except number of meals, showed significant interactions between group (robust or sensitive) and environment. Overall, the robust group as defined by production and thermoregulation traits showed smaller differences in all feeding behaviour traits values from TEMP to TROP than the sensitive group. Within environment, we found that in TEMP, robust and sensitive did not show significant differences for any feeding behaviour traits (P > 0.05), except feeding rate which was higher in the sensitive group (31.6 vs. 34.8 g/min, P < 0.001). Conversely, in TROP, group differences were observed for all feeding behaviour traits (P < 0.01), except feeding rate (P = 0.93).

**Table 2:** Least squares means effect of robust and sensitive groups, production environments sire family and sex on feeding behaviour and performance traits.

Environment	Temperate		Tropical		RSD^1^	Significant effects^2^
Group	Robust	Sensitive	Robust	Sensitive		
No. of pigs	179	194	216	179		
**Feeding behaviour traits**						
ADFI^3^, g/d	2,207^a^	2,321^a^	1,886^b^	1,729^c^	410	SF*, E***, S***, Pe***, GxE***
Daily eating time, min	78.2^a^	75.2^a^	70.0^b^	61.8^c^	15.7	SF***, E***, S***, Pe***, G***, GxE*
Number of meals	6.2^a^	6.3^a^	7.4^b^	7.7^b^	1.7	SF***, E***, S**, Pe***
Feeding rate, g/min	31.6^a^	34.8^b^	29.0^c^	29.5^c^	7.4	SF*, E***, Pe***, G**, GxE*
Meal size, g	375^a^	392^a^	273^b^	232^c^	91	SF***, E***, S^†^, Pe***, G^†^, GxE***
Meal duration, min	13.2^a^	12.5^a^	10.1^b^	8.2^c^	3.0	SF***, E***, Pe*, G***, GxE**
**Performance traits**						
Final BW^4^, g	101.3^a^	102.8^a^	87.8^b^	81.6^c^	9.0	SF***, E***, S***, G***, GxE***
ADG^5^, g/d	811^a^	817^a^	772^b^	715^c^	82	SF***, E***, S***, G***, GxE***
BFT^5,^ mm	19.5^a^	21.5^b^	16.0^c^	15.2^d^	2.7	SF***, E***, S***, Pe**, G**, GxE***
FCR^7^ kg feed,/kg BW gain	2.7^a^	2.8^a^	2.5^b^	2.4^b^	0.5	SF^†^, E***, S*, Pe***, GxE*
RFI^8^, g/d	64.6^a^	77.4^a^	6.2^a^	-23.2^b^	370	E**, Pe***

^a-d^Within a row, means with a different superscript letter differ, P < 0.05.

^1^Residual Standard Deviation.

^2^From an analysis of variance with a linear model including the effects of Sire Family (SF) within Group, Environment (E), Sex (S), Period of feed intake recording (Pe), Group (G) and their interactions as fixed effect. Batch within environment was significant for all traits and is not reported in the table. Statistical significance: ***P < 0.001, **P < 0.01, *P < 0.05, ^†^P ≤ 0.10.

^3^ADFI = Average Daily Feed Intake.

^4^BW = Body Weight.

^5^ADG = Average Daily Gain between 11 and 23 weeks of age.

^6^BFT = Backfat Thickness at 23 week of age.

^7^FCR = Feed Conversion Ratio between 11 and 23 weeks of age.

^8^RFI = Residual Feed Intake between 11 and 23 weeks of age.

**Figure 4:**
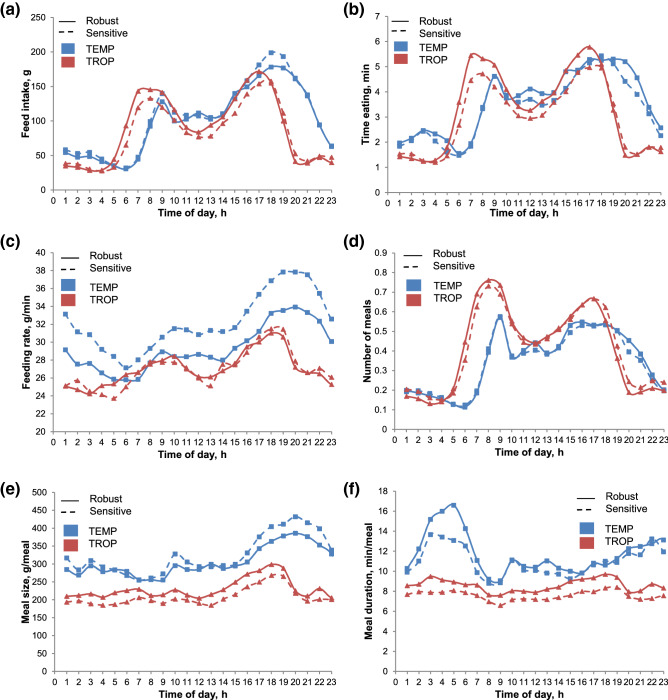
Hourly distribution of feeding parameters according to the robust/sensitive group: robust group (solid line), sensitive (dotted line) and production environment: temperate (red), tropical (blue). (**a**) Feed intake, (**b**) time feeding, (**c**) feeding rate, (**d**) number of meals, (**e**) feed intake per meal, (**f**) time per meal. In TEMP conditions: robust group, n = 179; sensitive group, n = 194. In TROP conditions: robust group, n = 216; sensitive group, n = 179.

The comparison of robust and sensitive groups for daily kinetics of FI and other feeding traits showed similar patterns of feeding behaviour within and between-environments (Fig. 4a–f). We found a significant interaction between hour, group and environment for all parameters (P < 0.05) but the main differences between the two groups were not observed in the daily dynamics of feeding traits, but in their values. Overall, the robust group had higher values in TROP than the sensitive group for all behaviour traits, except for feeding rate (Fig. 4c) and frequency of meals (Fig. 4d).

## Discussion

The current literature on the effect of the environment and more particularly on the effect of GxE interactions on feeding behaviour traits is scarce. With our experimental design in which contemporary half sib pigs were reared in two contrasting environments, we aimed at evaluating the effects of tropical production conditions on feeding behaviour traits, focusing on the interactions between sire family and environment (GxE) and how they relate to performance and thermoregulation traits.

The upper limit of thermoneutrality, in which no extra energy is used for thermoregulation, is considered to be around 25 °C for growing-finishing pigs^17,18^. In our study, temperature in TEMP varied daily on average between 24.7 and 25.7 °C, suggesting that pigs were reared closed to thermoneutral conditions. In TROP, daily temperature variation was more important, between 24.1 and 29.1 °C on average, with temperature being above 25 °C from 7:00 to 23:00. Therefore, animals in TROP needed to adjust for chronic HS (15 h out of 24 h) as well as 4 °C of temperature variation during the day. However, the effects of TROP climate on performance, thermoregulation and feeding behaviour traits are consistent with studies where animals were submitted to constant HS, suggesting that a 4 °C daily temperature variation does not modify the effect of HS on the different traits.

The effects of tropical climate on performance and thermoregulation traits have already been detailed in Rosé et al*.*^16^. Consistent with previous studies on HS, TROP environment reduced FI and performance traits^3^, but increased feed efficiency^3,17^ and body temperature traits (ST, RT)^2^. In the present study, we showed that TROP conditions also modifies feeding behaviour of the pigs: animals had slightly more meals per day but these meals were smaller both in duration and in size, resulting in lower ADFI and lower time spent eating per day.

The feeding pattern can be described as the combination of feeding behaviour traits that will allow to reach a given level of daily feed intake^18,19^. It is noteworthy that markedly different feeding patterns can give rise to the same daily feed intake^12,18^. Based on previous studies, four feeding patterns have been suggested in pigs: two are based on the frequency and size of meals: nibbler vs. meal eater, while the other two are based on the rate of feed intake: slow vs. fast eater^19^. Here, we showed that animals in TEMP conditions have a meal eater/fast eater pattern whereas animals in TROP adopted a nibbler/slow eater pattern. Previous studies showed that the environment modifies the feeding pattern and feeding behaviour^10^. Consistent with our study, high temperature has been shown to reduce meal size and duration^11,12,20,21^ and to increase meal frequency^12^. When comparing individual precision feeding and conventional diets under HS and thermoneutral conditions, Dos Santos et al. (2018)^22^ found that HS reduced time spent eating, meal size and feeding rate in both feeding systems. Effects of HS on feeding rate are not consistent between studies, probably due to differences in methodology and feeding rate being variable with age^8,23,24^, body size, breed^19^ and housing conditions^24^. The lower feeding rate observed in TROP could be explained by the lower BW in TROP compared to TEMP. Indeed, in agreement with previous results described in the literature^8,23,24^, we observed that feeding rate increased with age (result not shown), probably as a result of increased body size, including the capacity of the mouth and gastrointestinal tract filling. However, a direct effect of HS is the reduction of FI and as a consequence reduction of BW, therefore discriminating between a direct effect of HS and a consequence of reduced FI on feeding rate would necessitate to perform pair-feeding experiments. When comparing animals of similar BW, Renaudeau et al*.*^12^ found that Creole pigs had a lower feeding rate than Large White pigs, which may suggest that pigs adapted to the tropical climate (like the Creole breed) have a slower feeding rate, as observed in our study.

When investigating the effect of feed restriction on feeding behaviour, Carco et al*.*^25^ found that restrictively-fed animals ate faster, suggesting that feeding rate may reflect feeding motivation in pigs. This result suggests that TROP conditions, despite resulting in a moderate feed restriction (around 90% of ad libitum), reduce feeding motivation for the pigs. Recent studies on palatability in rodents and pigs suggest that longer meals could be associated with a higher hedonic response during feed intake^26,27^. With reduced meal duration in TROP compared to TEMP, animals may perceive less pleasure during feed intake, which would be consistent with reduced feeding motivation. One hypothesis could be that in TROP, feeding rate and thus, feeding motivation, are reduced as a way to reduce the thermic effect of feeding and thereby of metabolic heat production^28^ which would not be the case in TEMP were metabolic heat can be more easily dispersed. In accordance with this hypothesis, we found small but significant negative residual correlation between feeding rate and RT_19-23_ in TROP. However, it is noteworthy that due to the very large number of feeding behaviour observations, small correlations can be significant, despite an important part of the variation remaining unexplained.

Overall, residual correlations between thermoregulation and feeding behaviour traits were low and rarely significant. To our knowledge, data on phenotypic correlations between body temperatures and feeding behaviour is scarce. Low residual correlations between thermoregulation and production traits were obtained from the same data set^16^. In pigs, RT varies with ambient temperature and feed intake and follows a circadian pattern^29,30^. Therefore with one measurement of RT in the morning, we may limit our estimation of accurate correlations. It could also be that there is limited association between body temperature and feeding behaviour in our test conditions.

In the present study, we also investigated how the modification of feeding pattern with the production environment was related to performance traits. Consistent with previous studies^7,31,32^, we found that in TEMP, ADG was most highly correlated with ADFI, meal size and feeding rate. Therefore, it is likely that pigs with a meal eater and fast eater pattern would have some productivity advantages with higher FI and higher growth rate. Similar conclusions were suggested in Fernandez et al*.*^19^ when they compared feeding patterns in four different breeds of pigs. In our study, we also found high residual correlations between BFT and ADFI, meal size and feeding rate, suggesting that the meal eater/fast eater pattern would also lead to higher fat deposition. Rauw et al*.*^8^ found in Duroc pigs that animals that ate faster grew fatter, and other studies suggest similar relationships^7,31^. In humans, several studies suggest that eating fast is related to higher body mass index^33^ and insulin resistance^34^, whereas eating slowly increases the anorexigenic gut peptide response^35^.

In our study, in TEMP, residual correlations between number of meals and performance traits were low whereas correlations with meal size and performance traits were medium to high. This result suggests that meal size rather than meal frequency is important for productivity. The low correlations of meal frequency with performance can be explained by an antagonistic effect of increasing meal frequency: on one hand, more meals per day may result in higher efficiency of amino acid utilization^36^ but on the other hand it may increase energy demands for maintenance with an increase of physical activity. Consistent with the latter, in both environments, measures related to feed efficiency, RFI and FCR, were correlated with meal frequency: pigs that eat more often were less efficient, suggesting higher maintenance costs. RFI and FCR were also positively correlated with other feeding parameters: ADFI, feeding rate, meal size and daily eating time. This is consistent with a study comparing feeding behaviour in a divergent line of pigs selected for RFI where they found that pigs from the low RFI line (which are more efficient) have less meals, lower ADFI, daily eating time and feeding rate^37^.

In previous studies on the same dataset, significant SFxE interactions were found for performance and thermoregulation traits^16,29,41^. In particular, re-ranking of performance between TEMP and TROP climate was observed, demonstrating that the best sires for production traits in TEMP would not be the best ones in TROP. With climate change, it becomes crucial to understand and take into account GxE interactions in selection programs^42,43^. Most breeding programs are now transnational and should aim at providing animals that perform in a variety of environments^44,45^. However, GxE has been considered as a source of inefficiency in breeding programs for a long time^46^ and although research on GxE interactions is increasing, data in pigs remain scarce. In particular, very few studies have focused on how GxE interactions may impact feeding behaviour, i.e. how different genotypes may modify their feeding behaviour in response to different environments. When studying overall feeding activity in the progeny from different sire breeds, Cross et al*.*^15^ found genomic variation and genetic markers for feeding activity changes induced by HS events. Consistent with these results, in our study, we found that all feeding behaviour traits show significant SF by production environment interactions.

Feeding behaviour directly affects feed intake and is therefore related to performance but it is also closely related to the individual adaptive capacity^47^ and more generally to the animal welfare^18^. Moreover, feeding behaviour traits are non-invasive measures and with the rapid development in monitoring technologies on farm, individual measures can now be recorded easily. Thus, compared to thermoregulatory traits that are difficult and costly to measure, feeding behaviour traits could be good candidates as proxies to evaluate heat tolerance.

For this purpose, we compared the feeding behaviour pattern of robust and sensitive families (defined according to performance and thermoregulatory traits) in TEMP and TROP conditions. Our results highlighted significant differences between robust and sensitive groups for all feeding behaviour traits, except meal frequency. For all traits, the robust group was also more robust regarding feeding behaviour traits, i.e. the robust group had a smaller change of trait values than the sensitive group.

Interestingly, when comparing the two groups intra-environment, feeding rate was the only feeding trait for which there was a difference between robust and sensitive groups in TEMP. In accordance with these results, the daily pattern of feeding traits was very similar between robust and sensitive families in TEMP and only differed for feeding rate. The sensitive group had a higher rate of feeding in TEMP compared with the robust group, whereas both groups had similar feeding rate in TROP. Feeding rate varies with age^8,23,24^, body size, breed^19^ and housing conditions^24^. No difference in final BW was observed between groups in TEMP, thus, reduced feeding rate of the sensitive group in TEMP cannot be explained by differences in BW. From these observations, we may suppose that robust SF would have a lower feeding rate, which would somehow provide better heat tolerance. One hypothesis could be that the slow eater pattern may be better adapted to the tropical production environment. To our knowledge, no studies have investigated the relationship between feeding rate and heat production and how it may relate to better heat tolerance. Nonetheless, as mentioned above, when compared to Large White pigs of similar BW, Creole pigs have lower feeding rate, which may suggest that reduced feeding rate could be an adaptation to HS^12^. Selection for high FI and high productivity has been suggested to confer higher sensitivity to stress^43^ and may have selected animals with a fast eater pattern. In line with the later hypothesis, we do observe positive residual correlation between feeding rate and feed efficiency measures (RFI and FCR) in both environments.

In conclusion, the present study demonstrates strong plasticity of feeding behaviour traits and overall feeding pattern of pigs in response to HS, impacting performance traits. Moreover, this is the first study on GxE interactions for the feeding behaviour of growing pigs genetically related in two contrasting environments. We found significant GxE interaction for all feeding behaviour traits and our results suggest that feeding rate may be a good candidate to evaluate heat tolerance in temperate conditions. The high feeding rate observed in the sensitive group in TEMP conditions reinforced the conclusions of previous studies that the best sires in TEMP are not necessarily the best ones in TROP. Further studies involving genetic and genomic analysis for deciphering chromosomal regions related to feeding behaviour are in course and should provide better understanding of the relationships between feeding behaviour, heat tolerance and performance traits.

## Methods

This study was carried out in compliance with the ARRIVE guidelines^48^. All measurements and observations on animals were performed in accordance with relevant guidelines and regulations on animal experimentation and ethics (CE2012-9 from the Animal Care and Use Committee of Poitou–Charentes and 69-2012-2 from the Animal Care and Use Committee of French West Indies and Guyana) and the experimental protocol was approved by the French Ministry of Agriculture and Fisheries (authorization number: 17015 and 971-2011-03 7704, respectively) under the direction of Y. Billon (INRAE-GenESI) and J. Fleury (INRAE-PTEA).

### Experimental design

Data used in the present study were obtained in a backcross (BC) population (3/4 Large White (LW) and ¼ Creole (CR) breed) initially designed to examine the genetic background of heat tolerance in growing pigs. Detailed description of the experimental design has been given in two other studies^16,41^. Briefly, data were collected from April 2013 to October 2014 in the closed facilities of the INRAE experimental farm located in TEMP (INRAE experimental facility Le Magneraud, GenESI, Surgères, Charentes, France https://doi.org/10.15454/1.5572415481185847E12) and in the semi-open front unit of the INRAE experimental farm located in TROP (INRAE experimental facility PTEA, Petit-Bourg, Guadeloupe, France). In TEMP conditions, the closed facilities were temperature-controlled through a ventilation system and artificial light was provided from 8:00 to 18:00. In TROP conditions, the semi-open front building was subjected to the outside temperature and light variation (average light period from 6:00 to 18:00).

A total of 634 BC pigs from 60 LW sows (raised in 11 contemporary batches) and 662 BC from 70 LW sows (12 batches) were obtained in TEMP and TROP conditions, respectively. Backcross growing pigs were connected via the same 10 F1 CR × LW boars used to sire genetically related LW sows (same sires and maternal grand-sires) in the two farms. The test was set during the growing period from 11 to 23 weeks of age, using the same protocol in TEMP and TROP conditions. In both farms, animals were housed in closed growing pens of similar size (5.7 × 2.7 m), equipped with similar automatic feeders, and with the same number of animals per pen. Pens of 10 pigs of the same sex (females or castrated males) were constituted at 10 weeks of age, and evaluated after 5 days of adaptation to the new environment. Pigs were fed *ad-libitum* with a commercial feed formulated with the same nutritional characteristics in the two farms (15.7 MJ DE/kg, 170 g CP/kg of Dry Matter). Animals had free access to water.

### Phenotypic recording

Room ambient temperature (T) and relative humidity (RH) were recorded during the whole duration of the test period. In TEMP conditions, these climatic parameters were obtained every 5 min in the closed experimental facilities using a stand-alone USB data logger (EL-USB-2 + ; DATAQ Instruments, Inc., Akron, OH) located at the center of the room. In the TROP conditions, the semi-open building was equipped with a Campbell weather station (Campbell Scientific Ltd., Shepshed, UK) continuously recording ambient T and RH (1 measurement every 30 min) in each room of the experimental farm.

All animals were weighed every two weeks, from 11 (BW_11_) to 23 (BW_23_) weeks of age. ADG was calculated between weeks 11 and 23. BFT was measured on week 23 as the average of six ultrasonic measurements (Agroscan, E.C.M., Angoulême, France) at 6 different sites, measured directly above the point of the elbow, last rib (P2 site) and last lumbar vertebra locations, respectively, and taken 5 cm off the midline on each side of the pig. Single place electronic feeders (ACEMA 128, ACEMO, Pontivy, France) were available to record individual feed intake during the test. Rectal temperature (RT) was measured at week 19, 21, and 23, and skin temperature (ST) was measured at week 23. Digital thermometers (Microlife Corp., Paris, France) were used to measure RT, and ST was measured on the back at P2 site using a skin surface thermocouple probe (type K, model 88002 K-IEC; Omega Engineering Inc., Stamford, CT) connected to a microprocessor-based handheld thermometer (model HH-21; Omega Engineering Inc.). The RT and ST measurements were performed on unrestrained animals and with a minimum of stress during the weighing events in the morning.

Due to the experimental limitations and to maximize the number of pigs with feeding measurements, during the 12 weeks of test, pigs had access to automatic feeders for three periods of two weeks, which alternated with periods of two weeks fed with conventional collective feeders. Hence, for half of the pigs (defined as period 1), feeding behaviour data was available for week 11–12, week 15–16 and week 19–20 and for the other half (defined as period 2), data was recorder for week 13–14, week 17–18 and week 21–22. During the remaining test weeks, pigs had free access to conventional collective feeders. Pigs switched from one feeding system to the other on the Monday after weighing. All feed dispensers were calibrated at the start of each replicate using a 1-kg test weight. Each feeding stall allows access to only one pig at a time. After each visit to the feeder, the identity of the animal (via the ear-tag transponder), the feeder entry and exit times and the amount of feed consumed were registered and stored in a central equipment memory.

### Calculations

A temperature–humidity index (THI) was calculated for each day based on the following formula proposed by the National Oceanic and Atmospheric Administration (NOAA^49^; cited by Zumbach et al*.*^50^: THI = T − (0.55 − 0.0055 × RH) × (T − 14.5), in which T is the average daily T (°C) and RH is the average relative humidity.

ADFI was calculated from data collected by the electronic feed dispensers by averaging daily feed intake records of the 6 week available for each pig in either period 1 or period 2. We calculated FCR as ADFI divided by ADG. RFI was computed for each animal as the deviation between ADFI and ADFI predicted by a regression of ADFI on ADG between 11 and 23 weeks of age, BFT, and the average metabolic body weight during the test, as proposed by Rose et al*.*^16^.

Feeding behaviour traits were calculated from data collected by the electronic feed dispensers of the 6 weeks available for each pig in either period 1 or period 2. Schulze et al*.*^51^ recommended to exclude the 2 first days of recording to obtain reliable feed intake information. In a preliminary analysis, it has been shown that only the exclusion of the day when the animals switched between single and collective feeding systems is needed because readaptation to electronic feeders was not necessary. Consequently, feeding behaviour traits were estimated with exclusion of the days of switching between electronic feeders and conventional feed dispenser. Visits to the feeder where feed consumption was zero were removed from the dataset. The final dataset consisted of 1,728,688 visit records reporting animal identity, date, entering and exiting times, and feed consumption per visit, which were collected throughout the experiment from the 1,296 animals in the two farms (634 in TEMP and 662 in TROP). In order to allow comparison with results from other studies, successive feeder visits were grouped into the same meal using a meal criterion. Visits separated by intervals shorter than the meal criterion were considered to be part of the same meal^23,52^. In both production conditions, if time at feeder exceeded 5 min, the meal criterion duration no longer affected the number of meals per day. From this result, the adopted meal criterion for the present study was 5 min and this value was chosen for further calculation of daily eating behaviour traits. These traits were the daily feed intake (g), daily eating time (i.e. total duration of all feeding bouts, min), feeding rate (i.e. daily feed intake/daily eating time, g/min), daily number of meals, average feed intake per meal (meal size, g), and average feeding time per meal (meal duration, min). For each environment, the circadian rhythm of the 6 feeding behaviour traits was studied by summing the data for each hourly interval (from 0000 to 2400 h) for each pig and each day.

### Statistical analysis

As feeding behaviour data was recorded daily for individual pigs, each trait was averaged by pig and was analysed using linear models (GLM procedure; SAS version 9.4; SAS Inst. Inc., Cary, NC) with the fixed effects of the environment (TEMP vs. TROP), sex (female vs. castrated male), batch within environment (11 in the TEMP environment and 12 in the TROP environment), SF (10 families), recording period (2 periods) and the following interactions : SF × environment and sex × environment as main effects. Data on production traits and thermoregulatory traits was analysed following the same model^16^.

Least squares means of the effects were computed, and the differences between the effect levels were tested with a Tukey test. Within each environment, Pearson correlations between traits were computed from residuals of these linear models to adjust the data for the fixed effect of the models.

The G × E interactions were assessed by studying the least squares means from the previous linear models when the interaction between sire family and environment was significant (P < 0.05). Assessment of robustness and sensitivity between the 10 sire families was performed according to Rosé et al*.*, (2017)^16^ based on production (BW at 23 weeks, BFT at 23 weeks, ADG between 11 and 23 weeks, ADFI between 11 and 23 weeks) and thermoregulatory traits (average RT at test weeks 19, 21, and 23). Based on the 5 traits of interest^16^ and consistent with results on the same dataset obtained by Dou et al*.* (2017)^41^, sire families 1, 2 and 7 were found to be the most robust sire families and formed the “robust group”. Conversely, sire families 5, 6 and 10 were the most sensitive sire families and were included in the “sensitive group". Feeding behaviour traits of the offspring of these six families were analysed again using linear models (GLM procedure; SAS version 9.4; SAS Inst. Inc., Cary, NC) with the fixed effects of the group (robust vs. sensitive), environment (TEMP vs. TROP), sex (female vs. castrated male), batch within environment (11 in the TEMP environment and 12 in the TROP environment), sire family within group (3 in each group), recording period (2 periods) and the following interactions : SF(G) × environment, sex × environment and group × environment as main effects.

The circadian rhythm of the feeding behaviour data was averaged by animal and by hour and analysed through a repeated measurement analysis of variance with a compound symmetry (CS) covariance structure (Mixed procedure, SAS version 9.4; SAS Inst. Inc., Cary, NC) with the fixed effects of the hour (24 h), environment (TEMP vs. TROP), sex (female vs. castrated male), batch within environment (11 in the TEMP environment and 12 in the TROP environment), sire family (10 families), recording period (2 periods) and the following interactions : hour × SF, hour × environment, SF × environment and hour × SF × environment as main effects. The circadian rhythm of the robust and sensitive groups was analysed using a similar model with the addition of the fixed effect of group (robust vs. sensitive) and its interaction with other effects as main effects.

### Ethical approval

All measurements and observations on animals were performed in accordance with relevant guidelines and regulations on animal experimentation and ethics (CE2012-9 from the Animal Care and Use Committee of Poitou–Charentes and 69-2012-2 from the Animal Care and Use Committee of French West Indies and Guyana) and the experimental protocol was approved by the French Ministry of Agriculture and Fisheries (authorization number: 17015 and 971-2011-03 7704, respectively) under the direction of Y. Billon (INRAE-GenESI) and J. Fleury (INRAE-PTEA).

## Data Availability

The datasets generated and analysed during the current study are available from the corresponding author on reasonable request.
